# Bioactivity Profiling of Coronarin A and Villosin Isolated From *Hedychium coronarium* J. Koenig: Acaricidal, Nematicidal, and Antifungal Potential

**DOI:** 10.1002/cbdv.202502523

**Published:** 2026-04-01

**Authors:** Riddhiman Lahiri, Ravendra Kumar, Sushila Arya, Alok Pratap Singh, Sakshi Negi, Shivang Joshi, Pooja Bargali, Suraj N. Mali, Shilpi Rawat, Mozaniel Santana de Oliveira, Prahlad Sarkar

**Affiliations:** ^1^ Department of Chemistry College of Basic Sciences and Humanities G.B. Pant University of Agriculture and Technology Pantnagar Uttarakhand India; ^2^ Department of Soil Science & Agricultural Chemistry Veer Chandra Singh Garhwali Uttarakhand University of Horticulture and Forestry Bhararisain Uttarakhand India; ^3^ Bio Resource Development Department Dabur Research & Development Centre, Dabur India Limited Ghaziabad Uttar Pradesh India; ^4^ School of Pharmacy D.Y. Patil University (Deemed to Be University) Navi Mumbai India; ^5^ Department of Plant Pathology College of Agriculture G.B. Pant University of Agriculture and Technology Pantnagar Uttarakhand India; ^6^ Laboratory of Pharmacology of Inflammation and Behavior Graduate Program in Pharmaceutical Sciences Institute of Health Sciences Federal University of Pará Belém Brazil; ^7^ Department of Agricultural Entomology Uttarbanga Krishi Viswavidyalaya Pundibari West Bengal India

**Keywords:** coronarin A, *Hedychium coronarium*, *Meloidogyne incognita*, *Oligonychus coffeae*, *Pestalotiopsis microspore*, villosin

## Abstract

This study explores the biocontrol potential of two labdane‐type diterpenes, coronarin A and villosin, isolated from the rhizomes of *Hedychium coronarium* J. Koenig. The compounds were structurally characterized using FTIR, UV‐Vis, HPLC, and mass spectrometry. Their bioactivities were evaluated against the red spider mite (*Oligonychus coffeae*), the root‐knot nematode (*Meloidogyne incognita*), and the fungal pathogen *Pestalotiopsis microspora*. Coronarin A exhibited superior nematicidal activity, with 82.05% juvenile mortality and significant egg hatching inhibition at 5.0 mg/mL. Villosin demonstrated stronger acaricidal efficacy, achieving 90.28% mite mortality at the same concentration. Moderate antifungal effects were observed, particularly with villosin. Molecular docking studies revealed strong binding affinities of both compounds to acetylcholinesterase (AChE) and GABA transaminase (GABA‐T), supporting their pesticidal mechanisms. ADMET analysis indicated favorable pharmacokinetic and toxicological profiles, highlighting their safety and potential for agricultural applications. This study highlights the safety and potential biopesticidal potential of the compounds, as evidenced by in vitro and in silico analyses; however, their suitability for agricultural applications will be evaluated through further field trials in future research.

## Graphical abstract

The graphical abstract illustrates the flowchart for the isolation and evaluation of bioactive compounds from Hedychium coronarium. Crude extracts were obtained via cold percolation from the aerial parts (flowers, leaves, and stems) using dichloromethane (DCM) and from the rhizomes using chloroform. Each extract underwent fractional separation by column chromatography, yielding two purified compounds through sequential elution. Structural elucidation of the isolates was achieved using multiple spectroscopic techniques, confirming their identities as coronarin A and villosin. Subsequently, these compounds were subjected to in vitro bioefficacy assays (nematicidal, acaricidal, and antifungal activities) and in silico analyses (molecular docking and ADMET profiling).

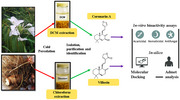

## 1 Introduction

Phytopathogenic pests such as the red spider mite of tea (*Oligonychus coffeae*), *Pestalotiopsis* spp. (fungi *causing grey‐blight disease in tea*), and root‐knot nematode (*Meloidogyne incognita*) represent significant threats to global tea productivity. The red spider mite, *Oligonychus coffeae* Nietner (Acarina: Tetranychidae), is among the foremost tea pests in India [1, 2, 3], and it causes the loss of up to 35%–40% of the crop [4, 5]. The tea plant's mature leaves are attacked by the veins and the mid‐rib; finally, the whole leaf is affected. In cases of severe infestation, the tender foliage may also become damaged [6, 7]. These pests compromise plant health through direct feeding damage, interference with nutrient uptake, and increased vulnerability to abiotic stressors and secondary infections. The fungal pathogen *Pestalotiosis* spp. is a polyphagous necrotroph with a broad host range and mainly infects maintenance foliage, which results in the complete defoliation of tea bushes [8]. In India, this disease accounts for an estimated 17% reduction in crop yield [9], while in China, grey blight has led to economic losses of up to 50% [10]. Traditional control strategies rely heavily on synthetic pesticides; however, growing awareness of their ecotoxicological consequences, environmental persistence, and risks to human and animal health has spurred the search for safer, sustainable alternatives. In this context, phytochemicals offer a promising avenue for the development of biopesticides compatible with integrated pest management (IPM) frameworks [11, 12].

Secondary metabolites such as terpenoids, alkaloids, flavonoids, glycosides, and phenolics have been extensively documented for their broad‐spectrum bioactivities, including antifeedant, insecticidal, nematicidal, acaricidal, antifungal, antioxidant, and antimicrobial properties [13]. These compounds function through diverse mechanisms, including neurotoxicity, membrane disruption, and enzyme inhibition, thereby providing a robust biochemical foundation for biopesticidal action. Their biodegradability and lower non‐target toxicity further enhance their utility in sustainable agriculture [13].

In this context, *Hedychium coronarium* J. Koenig of the Zingiberaceae family has demonstrated significant potential as a source of bioactive phytochemicals with significant applications in agriculture and medicine. Its essential oils, particularly from rhizomes and aerial parts, are rich in volatile constituents such as linalool, α‐ and β‐pinene, β‐caryophyllene, 1,8‐cineole, and α‐terpineol [14, 15]. These bioactive compounds have demonstrated significant nematicidal, insecticidal, antifungal, larvicidal, and herbicidal properties against various pests, including *M. incognita, Aedes aegypti*, and *Stephanitis pyrioides* [13, 16, 17]. Additionally, non‐volatile phytochemicals such as hedychilactone A, coronalactoside I, and isolinariins have been isolated and studied for their pesticidal and antimicrobial properties [18, 19, 20].

Several species within the genus *Hedychium*, including *H. flavum*, *H. gardnerianum*, and *H. spicatum*, have previously been reported to contain biologically active compounds with potent bioactivities relevant to pest management [21, 22, 23].

In the present study, the phytochemical composition of *H. coronarium* was compared with existing literature to validate the presence of known constituents, which were subsequently isolated, purified, structurally characterized, and evaluated for their biopesticidal potential. The novelty of this work lies in the identification and functional characterization of isolated compounds exhibiting specific pesticidal activity, particularly against the red spider mite (*Oligonychus coffeae*) infesting tea plants, the root‐knot nematode (*Meloidogyne incognita*), and plant pathogenic fungi (*Pestalotiopsis* spp.), thereby reinforcing the potential of *H. coronarium* as a botanical source for eco‐friendly biopesticide development.

## 2 Results and Discussion

### 2.1 Structural Elucidation of Isolated Compounds

**FIGURE 1:**
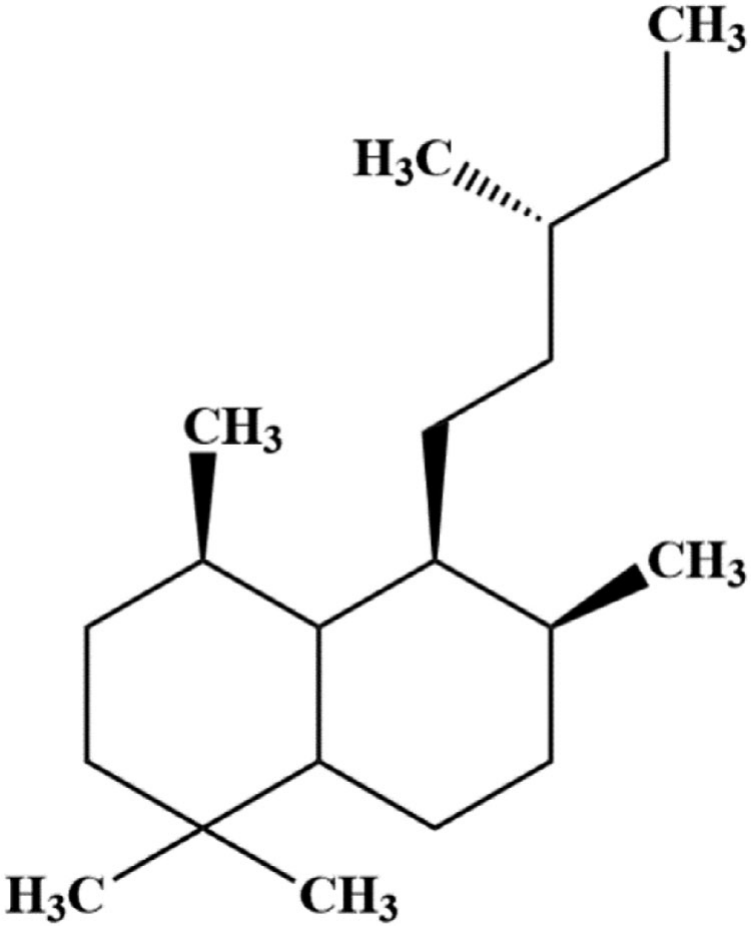
Basic structure skeleton of labdane‐type diterpenoids.

Diterpenoid derivatives represent a predominant focus of research within the genus *Hedychium*, owing to their distinctive chemical diversity and significant therapeutic applications among the broader class of terpenes. Numerous terpene compounds, particularly those classified as labdane‐type diterpenoids (Figure 1), have been isolated from *Hedychium coronarium*. Notable examples include hedycoronals, peroxycoronarin D, hedychicoronarin A, and hedychiumin, in addition to various sesquiterpenes and steroids [24].

**FIGURE 2:**
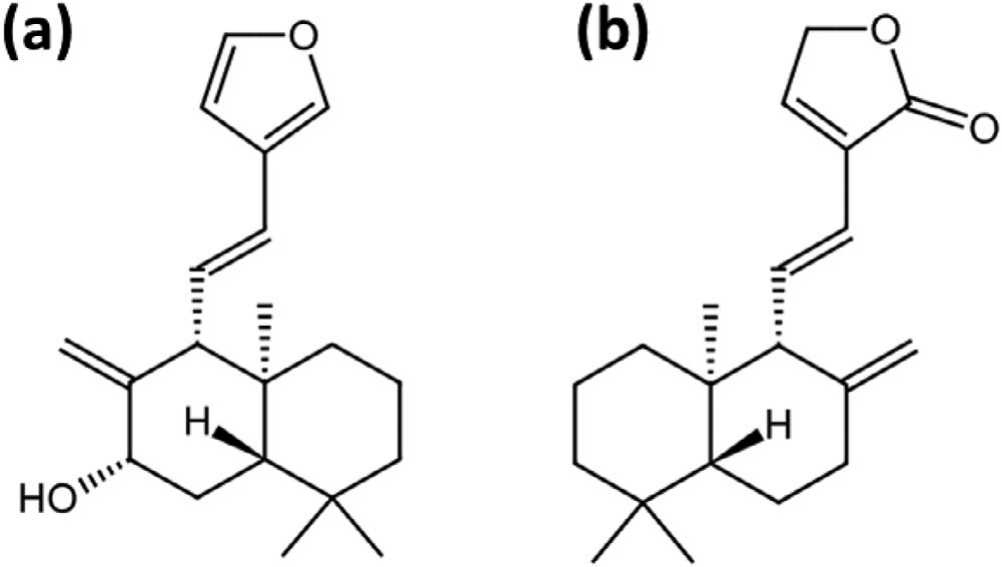
Structures of two isolated compounds: (a) compound 1; (b) compound 2.

Diterpenoids are characterized by a C20‐carbon skeleton, which is constructed from four isoprene units. Labdane‐related diterpenoids (LRDs) constitute a substantial subgroup, accounting for over 10% of all known terpenoids. These compounds typically feature a labdane‐type bicyclic core structure, though many exhibit more complex ring systems derived from the labdane skeleton, such as abietane, pimarane, kaurane, beyerane, atisane, cassane, stemodane, and manoyl oxide [25]. The biological activity of diterpenes varied significantly—the majority of diterpenes showed anticancer, cytotoxic, antifungal, and antibacterial properties [26, 27, 28]. Two isolated compounds, compound 1 (later identified as cornonarin A) and compound 2 (identified as villosin) (Figure 2), were identified using analytical results and published literature as mentioned in Table 1 and Table 2.

**TABLE 1:** Comparison between the analyzed data and data from published literature for confirmation of compound 1.

Analytical method	Current study	Literature data
Molecular formula	C_20_H_28_O_2_	C_20_H_28_O_2_
MW	300.4	300.44
[M + Na]^+^(m/z)	323.20	323
[M + H]^+^(m/z)	301.14	301
HPLC RT (min)	19.219	18–22
UV *λ*_max_ (nm)	250.5	250
FTIR (cm^−^^1^)	3400, 2925, 1700, 1600	3413, 2925, 1762, 1678

**TABLE 2:** Comparison between the analyzed data and data from published literature for confirmation of compound 2.

Analytical method	Current study	Literature data
FTIR C–H Stretch	2900–2950 cm^−^^1^	2900–2950 cm^−^^1^ (labdane diterpenes, villosin)
FTIR C═O Stretch	∼1700 cm^−^^1^	∼1700 cm^−^^1^ (labdane diterpenes, villosin)
FTIR C═C Stretch	∼1600 cm^−^^1^	∼1600 cm^−^^1^ (labdane diterpenes, villosin)
Mass [M + H]^+^	301.44	301 ([M + H]^+^, villosin)
Mass [2M + H]^+^/[2M + Na]^+^	618.79, 619.77	618–620 ([2M + H]^+^/[2M + Na]^+^, labdane diterpenes)

#### 2.1.1 Physical Property Assessment

The physical property assessment of compound 1 revealed a crystalline solid with a pale‐yellow color and crystalline texture when observed under magnification. The compound exhibited a melting point in the range of 105°C–108°C, consistent with literature reports for pure coronarin A (CID 34851535) [27]. In solubility tests, compound 1 showed moderate solubility in polar organic solvents such as ethanol and methanol, high solubility in dimethyl sulfoxide, acetone, and chloroform, and was practically insoluble in water.

Compound 2 (CID 16733738) was characterized as a yellow‐brown, resinous substance with a pronounced woody aroma. It exhibited significantly greater solubility in methanol, ethanol, and chloroform while remaining insoluble in water.

#### 2.1.2 Spectroscopic Analysis

**FIGURE 3:**
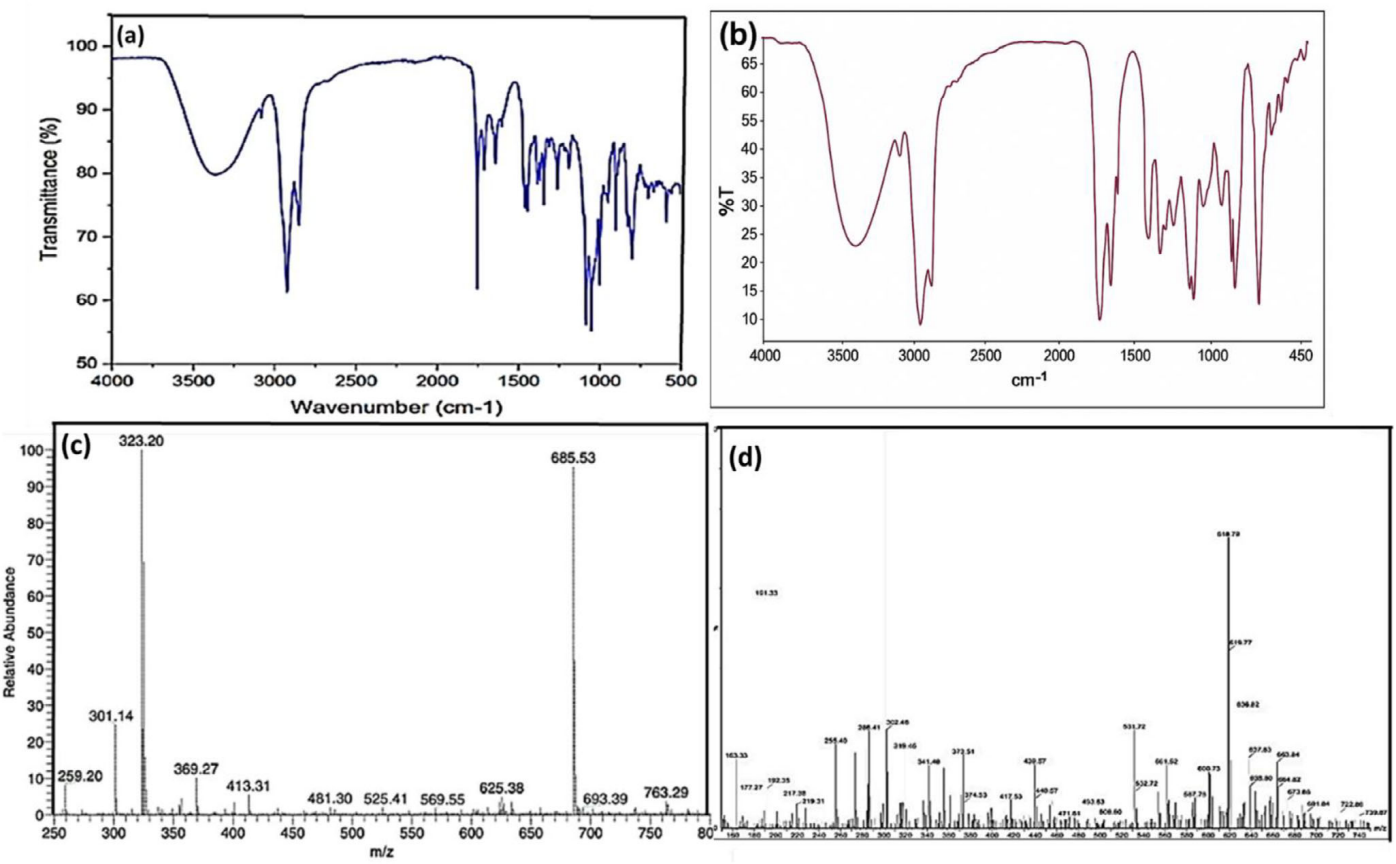
FT‐IR spectra for (a) coronarin A and (b) villosin; MS spectrogram for (c) coronarin A and (d) villosin.

**FIGURE 4:**
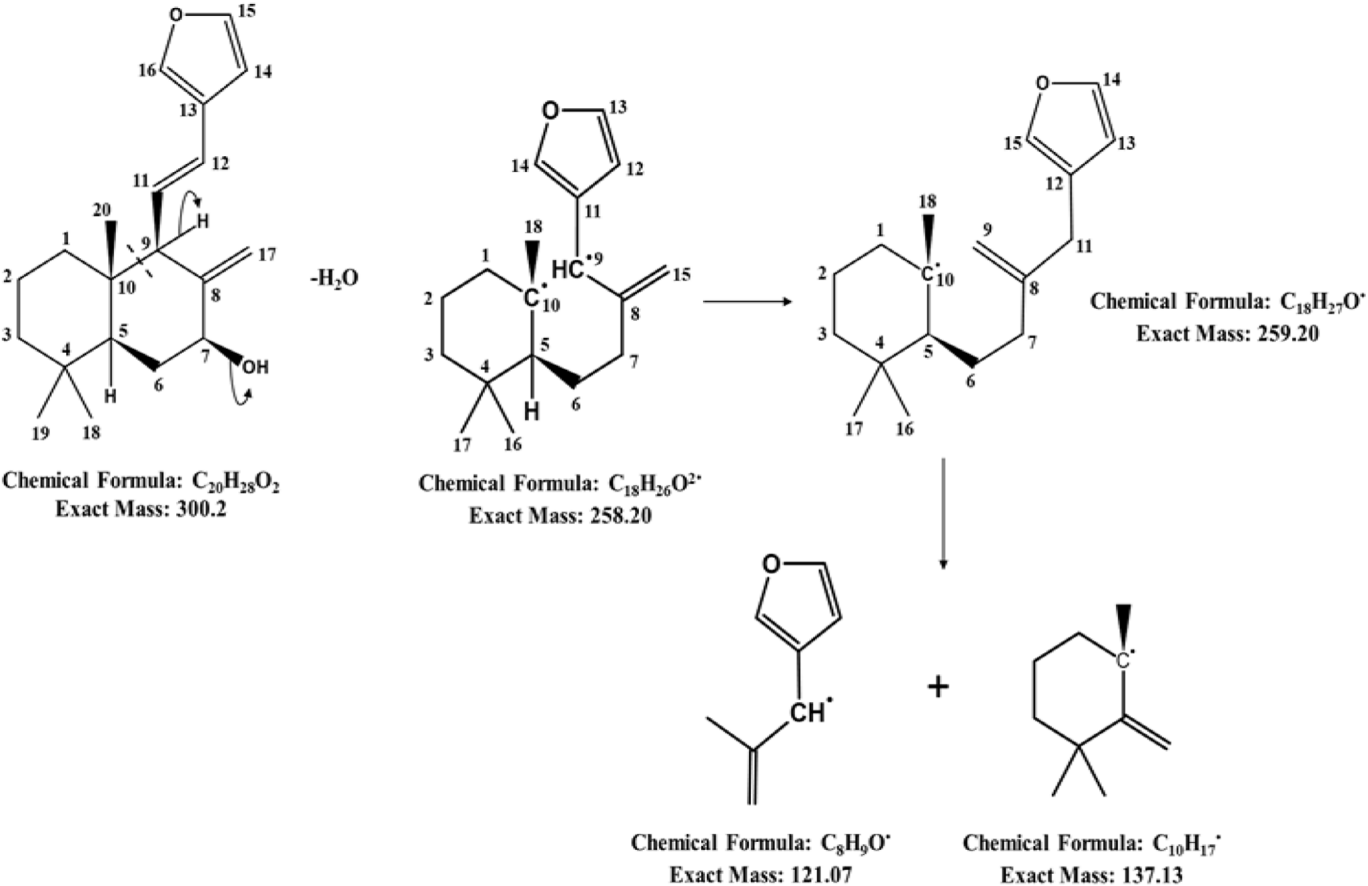
Possible mass fragmentation of coronarin A [27].

**FIGURE 5:**
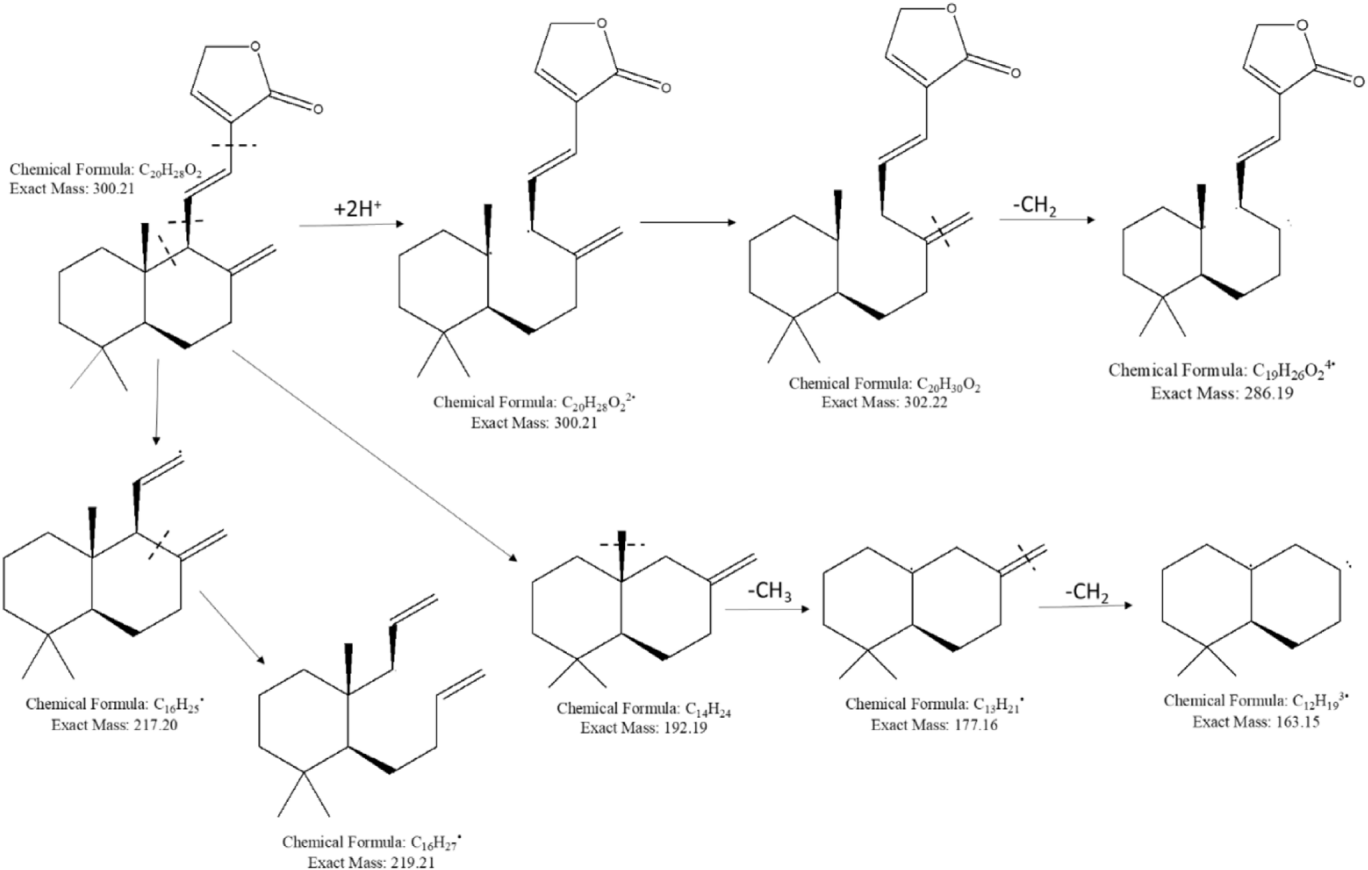
Possible mass fragmentation of villosin [27].

Also, the FTIR spectrum of compound 1 shows characteristic absorptions as a broad O‐H stretch (∼3400 cm^−^^1^), a C–H stretch (2900–3000 cm^−^^1^), a strong C═O (∼1700 cm^−^^1^), and a C═C (∼1600 cm^−^^1^), matching the published IR profiles for coronarin A and other labdane diterpenes from *Hedychium coronarium*. These features confirm the presence of hydroxyl, carbonyl, and conjugated alkene functionalities typical of coronarin A [27, 29]. The observed molecular ion peaks for compound 1, at m/z 323.20 ([M + Na]^+^) and 301.14 ([M + H]^+^), match the expected values for coronarin A (C_20_H_28_O_22_, MW = 300.44), as reported [27]. The presence of a dimeric sodium adducts at m/z 685.53 ([2M + Na]^+^) further confirms the labdane diterpene structure [27, 29]. Also, the FTIR spectrum of compound 2 reveals a broad O–H stretch around 3400 cm^−^^1^, C–H stretching at 2900–2950 cm^−^^1^, a strong C═O stretch near 1700 cm^−^^1^, and C═C stretching at 1600 cm^−^^1^. These features are characteristic of labdane diterpenes and have been specifically reported for villosin and related compounds as shown [30]. The mass spectrum of compound 2 shows a dominant [M + H]^+^ peak at m/z 301.44, which corresponds to the molecular ion of villosin (C_20_H_28_O_2_; MW 300.44), along with prominent dimeric peaks at m/z 618.79 and 619.77, consistent with [2M + H]^+^ and [2M + Na]^+^ adducts. These features are characteristic of labdane diterpenes in ESI‐MS and are well‐documented for villosin and related compounds [30]. FT‐IR spectra and MS spectrograms for coronarin A and villosin are shown in Figure 3. Figure 4 and Figure 5 illustrates the possible mass fragmentation of coronarin A and villosin.

#### 2.1.3 HPLC and UV Data

For compound 1, HPLC retention time (19.219 min) and UV *λ*_max_ (250.5 nm) are in excellent agreement with literature values (retention times 18–22 min, *λ*_max_ ∼250 nm) for coronarin A analyzed on C‐18 columns using acetonitrile‐water mobile phases. This reproducibility across independent studies confirms the reliability of these markers for identification [27, 29].

**FIGURE 6:**
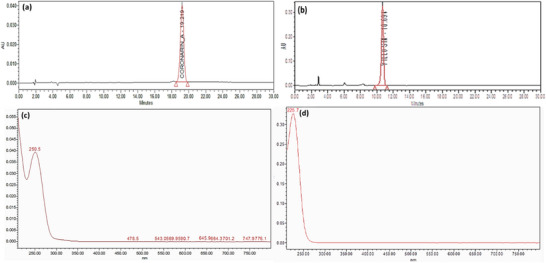
HPLC chromatogram for (a) coronarin A, *R*_t_: 19.219 min; (b) villosin, *R*_t_: 10.694 min; and UV scan profile of (c) coronarin A, *λ*_max_: 250.5 nm; (d) villosin, *λ*_max_: 225.7 nm.

Similarly, for compound 2, HPLC retention time (10.694 min) and UV *λ*_max_ (225.7 nm) are in excellent agreement with literature values (retention times 18–22 min, *λ*_max_ ∼220 nm) for villosin analyzed on C‐18 columns using acetonitrile‐water mobile phases. This reproducibility across independent studies confirms the reliability of these markers for identification [30]. Figure 6 represents the HPLC chromatogram and UV scan profile for coronarin A and villosin.

### 2.2 In Vitro Biological Activities

#### 2.2.1 Acaricidal Activity

The findings from the leaf disc assay [31, 32] revealed that among the two isolated plant‐derived compounds, villosin exhibited superior acaricidal efficacy at a concentration of 5.0 mg/mL, demonstrating the highest mortality even after 24 h of exposure (Table 3). After 48 h, both compounds, coronarin A and villosin, achieved mortality rates exceeding 80% at the same concentration, comparable to the synthetic acaricide fenpyroximate 5EC, which was effective at a significantly lower concentration of 0.5 mg/mL. The data presented in Table 3 indicate that both isolates exhibited strong acaricidal activity in a concentration‐ and time‐dependent manner. From probit analysis we can see coronarin A demonstrate higher LC_50_ (0.969 mg/mL) at 48 h. Notably their efficacy increased with prolonged exposure, and with substantial mortality observed after 48 h, suggesting that at higher concentrations, both compounds possess acaricidal potential comparable to that of the commercial standard fenproxymate. Further enhancement of efficacy by developing an acceptable formulation will be a continuation of our study. There is no previously reported result of labdane diterpenoids against red spider mites of tea. The anti‐insecticidal activities of the napelline‐type diterpenoid alkaloid C_20_‐diterpenoid alkaloid were evaluated against two‐spotted spider mites and showed moderate contact toxicity, with LC_50_ value of 0.95 ± 0.23 mg/mL [33]. This study highlights the potential use of diterpenoid alkaloids as natural plant‐derived pesticides for the management of red spider mites.

**TABLE 3:** Acaricidal assay (showing percent mortality) of coronarin A and villosin.

Compounds	Concentration (mg/mL)	Percent egg hatching and exposure time (h)			
		24 h	LC_50_ (mg/mL)	48 h	LC_50_ (mg/mL)
**Villosin**	1.0	33.43 ± 1.401^g^	1.629	47.81 ± 1.418^g^	1.117
	3.0	69.20 ± 2.987^d^		74.33 ± 1.910^e^	
	5.0	85.91 ± 1.307^a^		90.28 ± 1.695^a^	
**Coronarin A**	1.0	45.17 ± 1.443^f^	1.153	51.33 ± 0.956^f^	0.969
	3.0	78.17 ± 1.457^c^		79.21 ± 1.601^d^	
	5.0	85.75 ± 2.725^b^		89.93 ± 1.156^b^	
**Fenpyroximate 5EC**	0.5 mL/L	78.2 ±3.33^c^		80.4 ± 3.45^c^

*Note*: Results are expressed as mean ± standard deviation (*n* = 3). Tukey's test (*p* < 0.05) indicates that there is no statistically significant difference between mean values in a column that are followed by the same letter.

#### 2.2.2 Nematicidal Activity

Based on the data presented in Table 4 and Table 5, both Coronarin A and Villosin exhibited significant nematicidal activity, as demonstrated by their effects on nematode mortality and egg hatching inhibition. In the nematode mortality assay (Table 4), both compounds showed a concentration‐ and time‐dependent increase in percent mortality. At the highest tested concentration (5.0 mg/mL), coronarin A resulted in the greatest nematode mortality rates, achieving 64.33% at 24 h, 78.45% at 48 h, and 82.05% at 72 h. Villosin also displayed substantial nematicidal activity at 5.0 mg/mL, with mortality rates of 64.00%, 72.41%, and 76.33% at 24, 48, and 72 h, respectively. Lower concentrations of both compounds resulted in reduced mortality, but the trend of increasing mortality with both concentration and exposure time was consistent. The positive control, NIMITZ (0.5 mg/mL), exhibited the highest efficacy, with mortality rates exceeding 98% at all times.

**TABLE 4:** Nematode mortality assay (percentage mortality) of coronarin A and villosin.

Compounds	Concentration (mg/mL)	Percent mortality (%) with exposure		
		24 h	48 h	72 h
**Villosin**	1.0	41.00 ± 1.732^g^	48.33 ± 2.312^g^	54.44 ± 1.709^g^
	3.0	51.67 ± 2.309^e^	64.04 ± 1.738^e^	66.36 ± 2.211^e^
	5.0	64.00 ± 0.000^c^	72.41 ± 1.224^c^	76.33 ± 2.021^c^
**Coronarin A**	1.0	47.67 ± 1.667^f^	52.31 ± 0.011^f^	55.05 ± 0.681^f^
	3.0	55.67 ± 0.333^d^	68.01 ± 0.237^d^	72.88 ± 1.703^d^
	5.0	64.33 ± 1.453^b^	78.45 ± 0.006^b^	82.05 ± 0.602^b^
**NIMITZ**	0.5	98.00 ± 0.81^a^	98.00 ± 0.81^a^	99.00 ± 0.00^a^

*Note*: Results are expressed as mean ± standard deviation (*n* = 3). Tukey's test (*p* < 0.05) indicates that there is no statistically significant difference between mean values in a column that are followed by the same letter.

**TABLE 5:** Percent egg hatching inhibition against *M. incognita*.

Compounds	Concentration (mg/mL)	Percent egg hatching and exposure time (h)		
		24 h	48 h	72 h
**Villosin**	1.0	30.00 ± 2.64^a^	36.00 ± 3.46^a^	43.33 ± 2.309^a^
	3.0	22.67 ± 2.51^c^	29.67 ± 1.53^c^	37.00 ± 1.27^b^
	5.0	13.67 ± 2.08^e^	22.00 ± 3.61^d^	34.67 ± 1.15^d^
**Coronarin A**	1.0	25.67 ± 5.03^b^	30.67 ± 4.72^a^	36.00 ± 2.65^c^
	3.0	19.67 ± 2.08^d^	20.67 ± 2.08^e^	23.67 ± 4.51^e^
	5.0	9.670 ± 2.08^f^	14.67 ± 2.52^f^	20.00 ± 2.00^f^
**NIMITZ**	0.5	5.560 ± 1.23^g^	8.330 ± 1.50^g^	10.11 ± 1.53^g^

*Note*: Results are expressed as mean ± standard deviation (*n* = 3). Tukey's test (*p* < 0.05) indicates that there is no statistically significant difference between mean values in a column that are followed by the same letter.

In the egg hatching inhibition assay (Table 5), coronarin A and villosin both significantly inhibited egg hatching in a concentration‐ and time‐dependent manner. At 5.0 mg/mL, Coronarin A showed the strongest inhibition, with only 9.67% egg hatching at 24 h, decreasing further to 20.00% at 72 h. Villosin at the same concentration resulted in 13.67% egg hatching at 24 h and 34.67% at 72 h. Lower concentrations of both compounds were less effective, but still demonstrated substantial inhibitory effects compared to the control. Nimitz again served as a strong positive control, with egg hatching rates below 11% at all time points.

Overall, these results indicate that both coronarin A and villosin possess potent nematicidal activity, with coronarin A generally exhibiting greater efficacy than villosin in both nematode mortality and egg hatching inhibition assays. Previously reported diterpenoids isolated from *Hedychium* spp. root extract have been observed to have potent nematicidal activity. The nematicidal activity of these diterpenoids was as follows: isolinariin A (71.33% mortality) > coronalactoside I (69.00%) > hedychilactone A (68.33%) > (*E*)‐labda‐8(17), 12‐dien‐15(16)‐olide (66.33). The IC_50_ values of isolated compounds against the egg hatching process of *M. incognita* were recorded in the order of coronalactoside I (2.06 ± 0.04 µg/mL) > hedychilactone A (2.79 ± 0.02 µg/mL) > (*E*)‐labda‐8(17), 12‐dien‐15(16)‐olide (3.53 ± 0.05 µg/mL) > isolinariin A (3.84 ± 0.29 µg/mL) [28].

#### 2.2.3 Anti‐Fungal Activity

**FIGURE 7:**
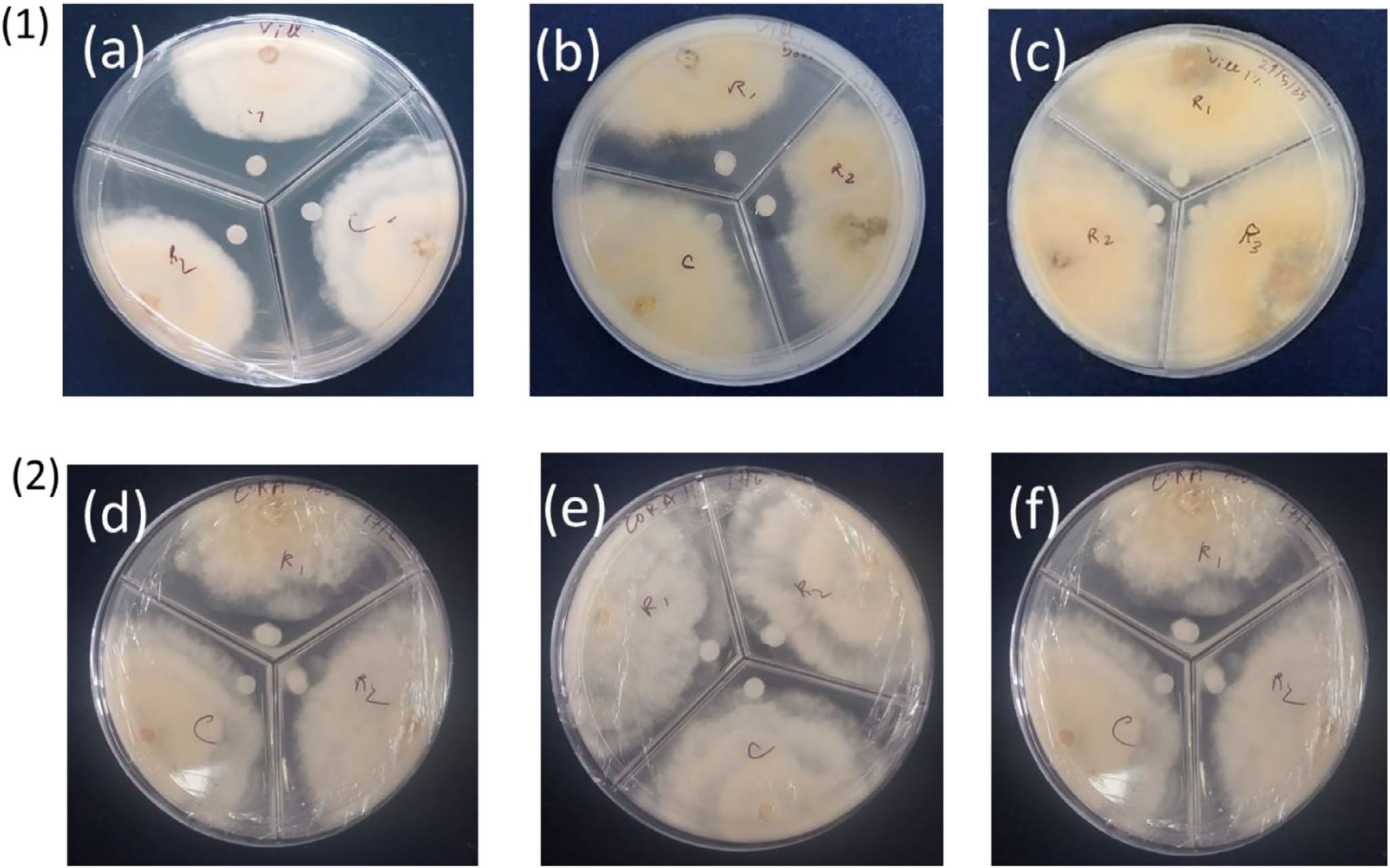
Plates showing antifungal activity of (1) villosin at (a) 40 µL/mL, (b) 20 µL/mL, and (c) 10 µL/mL; and (2) coronarin A at (a) 40 µL/mL, (b) 20 µL/mL, and (c) 10 µL/mL.

Villosin and coronarin A demonstrated concentration‐dependent antifungal activity against the tested fungal species, as reflected by their respective IC_50_ values of 19.9 mg/mL and approximately 65.25 mg/mL. Villosin consistently exhibited greater inhibitory potency than coronarin A at all concentrations evaluated. These results, detailed in Table 6 and visually represented by inhibition zones in Figure 7, indicate that villosin is a more effective antifungal agent under the conditions tested. Villosin and coronarin A have structures similar to that of coronarin D, which has been shown to be effective against *C. albicans*, with MIC and MFC of 2.0 and 4.0 mg/mL, respectively [33]. We can observe that the increased activity of villosin may be due to the presence of an additional lactone moiety, similar to coronarin D.

**TABLE 6:** Antifungal activity assay (showing % inhibition of mycelial growth) of villosin and coronarin A.

Compounds	Concentration (mg/mL)	% Inhibition
**Villosin**	10.0	3.33 ± 0.00^d^
	20.0	16.66 ± 2.71^c^
	40.0	28.00 ± 1.87^b^
**Coronarin A**	10.0	3.22 ± 0.00^d^
	20.0	4.995 ±2.35^d^
	40.0	10.34 ± 2.87^cd^
**Carbendazim**	1.0	64.5 ± 2.303^a^

*Note*: Results are expressed as mean ± standard deviation (*n* = 3). Tukey's test (*p* < 0.05) indicates that there is no statistically significant difference between mean values in a column that are followed by the same letter.

Some previously reported similar diterpenoids isolated from *Hedychium* spp. root extracts have shown bioactivity in antifungal assays against *F. oxysporum* and *C. lunata*, with inhibition percentages in the following order: hedychilactone A (98.14%) > (*E*)‐labda‐8(17), 12‐dien‐15(16)‐olide (97.77%) > coronalactoside I (94.07%) > isolinariin A (93.70%) and (*E*)‐labda‐8(17), 12‐dien‐15(16)‐olide (98.88%) > hedychilactone A (97.77%) > isolinariin A (90.81%) > coronalactoside I (89.25%), respectively [28, 33].

### 2.3 In Silico Study

#### 2.3.1 Molecular Docking Study

The docking studies (Table 7) revealed that villosin (–10.0 kcal/mol) and coronarin A (–9.7 kcal/mol) exhibit stronger predicted binding affinities to acetylcholinesterase (AChE; PDB 6XYU) than the reference standards fenpyroximate (–9.0 kcal/mol) and fluensulfone (–7.9 kcal/mol), suggesting a potential for superior AChE inhibition and associated nematicidal or insecticidal activity. Fenpyroximate, however, demonstrated the most potent binding to GABA‐transaminase (–11.2 kcal/mol), which aligns with its established acaricidal mechanism of action. All tested compounds showed relatively weak binding to cyanase (5UK3) (–6.9 to –7.3 kcal/mol), indicating that this metabolic enzyme is unlikely to be a primary target. Supporting these in silico findings, bioassays demonstrated that villosin exhibited the highest acaricidal efficacy at 5.0 mg/mL, achieving substantial mite mortality within 24 h, while both villosin and coronarin A exceeded 80% mortality after 72 h, comparable to the synthetic acaricide fenpyroximate 5EC at a lower concentration (0.5 mg/mL). Both plant‐derived compounds displayed strong, concentration‐ and time‐dependent acaricidal and nematicidal activity, with increased efficacy observed at higher concentrations and longer exposures. In nematode mortality assays, coronarin A and villosin induced significant, dose‐dependent increases in mortality, with coronarin A achieving up to 82.05% and villosin 76.33% mortality at 72 h at 5.0 mg/mL, though both were less effective than the positive control NIMITZ (0.5 mg/mL), which consistently exceeded 98% mortality. Furthermore, both compounds significantly inhibited nematode egg hatching in a concentration‐ and time‐dependent manner, with coronarin A showing the most pronounced effect (as low as 9.67% hatching at 24 h and 20.00% at 72 h at 5.0 mg/mL), while villosin also demonstrated substantial inhibition. These results indicate that both coronarin A and villosin possess notable nematicidal and acaricidal potential, likely mediated by AChE inhibition, and their efficacy is comparable to commercial standards at higher concentrations, supporting their promise as botanical alternatives for pest management. A compound's binding affinity is mostly determined by its binding cavity and interactions with the different amino acids of the targeted protein. The formation of many hydrogen bonds between the ligand and amino acid residues in the protein's active site is often necessary for an efficient interaction **[** 34 **]**.

**TABLE 7:** Docking scores for ligand binding affinity with AChE and GABA‐T.

Compounds	AChE (6xyu) (kcal/mol)	GABA‐T (1OHW) (kcal/mol)	*Pseudopestalotiopsis* elongation factor 1 (EF1) (kcal/mol)
**Coronarin A**	−9.7	−8.6	−5.8
**Villosin**	−10.0	−9.2	−6.8
**Fenpyroximate** ^a^	—	−11.2	—
**Fluesulfone** ^b^	−7.9	—	—
**Carbendazim** ^c^	—	—	−4.6

^a^Standard for GABA‐T.

^b^Standard for AChE.

^c^Standard for EF1.

However, the interaction between elongation factor 1 (EF1) from *Pseudopestalotipsis* spp. and villsoin or coronarin A did not show any satisfactory results. But villosin shows interaction greater than carbendazim. But in vitro antifungal activity shows that positive control carbendazim has way more activity than villosin. This suggests that other factors may be responsible for the observed discrepancy.

**FIGURE 8:**
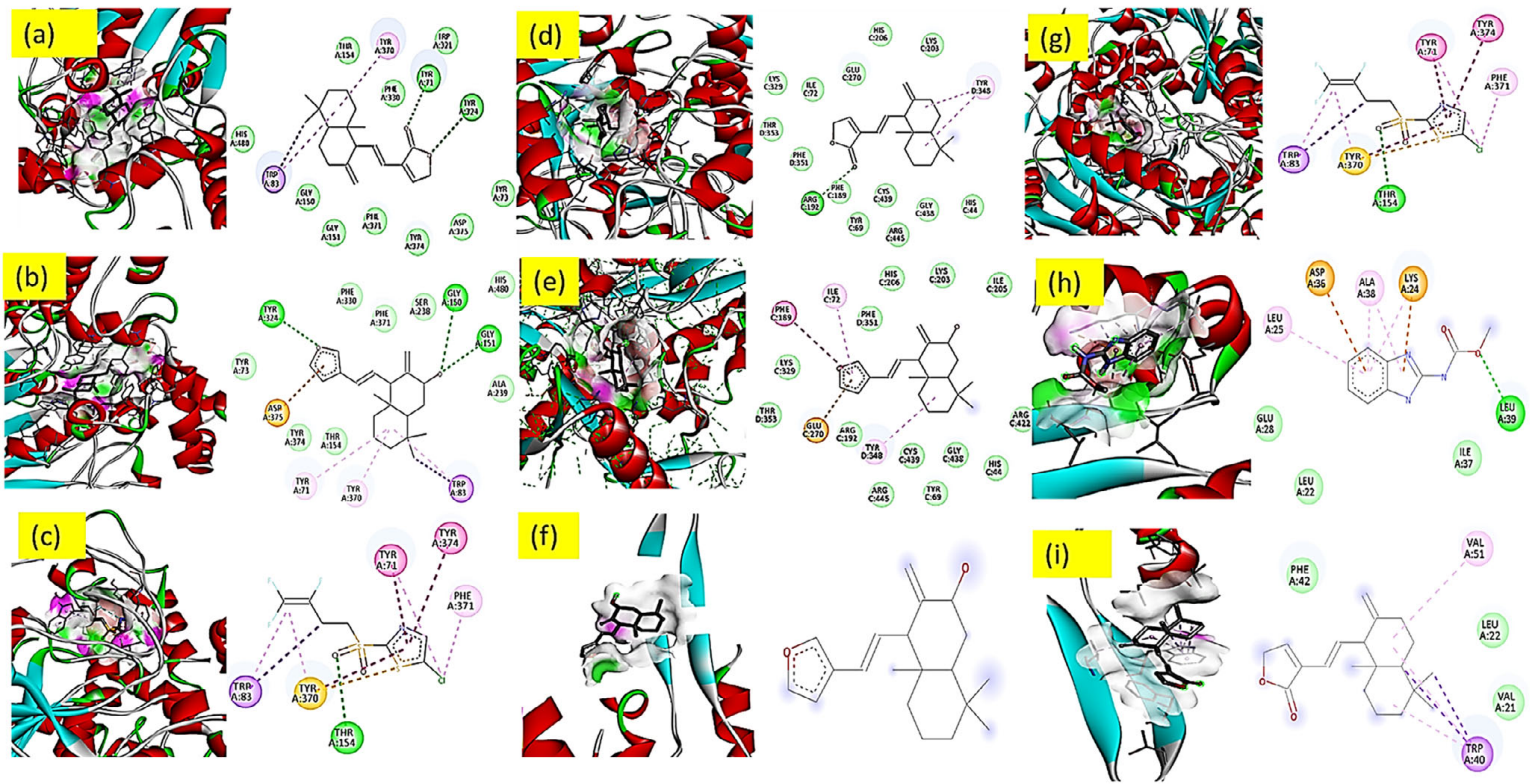
2D and 3D protein ligand interactions: (a) AChE‐Coronarin A, (b) AChE‐Villosin, (c) AChE‐fluesulfon, (d) GABA‐T–Villosin, (e) GABA‐T–Coronarin A, (f) GABA‐T–Fenpyroximate, (g) GABA‐T–Fenpyroximate, (h) EF1–Villosin, and (i) EF1–Coronarin A.

However, ligand‐protein interactions also include interactions between the target proteins' extra ligand‐amino acid residues. The results of molecular docking studies support the evaluation of in vitro biological tests. Several compounds in particular show promise for usage, according to the data. 2D and 3D interactions of ligands with selected target proteins are shown in Figure 8.

#### 2.3.2 ADMET Analysis

**FIGURE 9:**
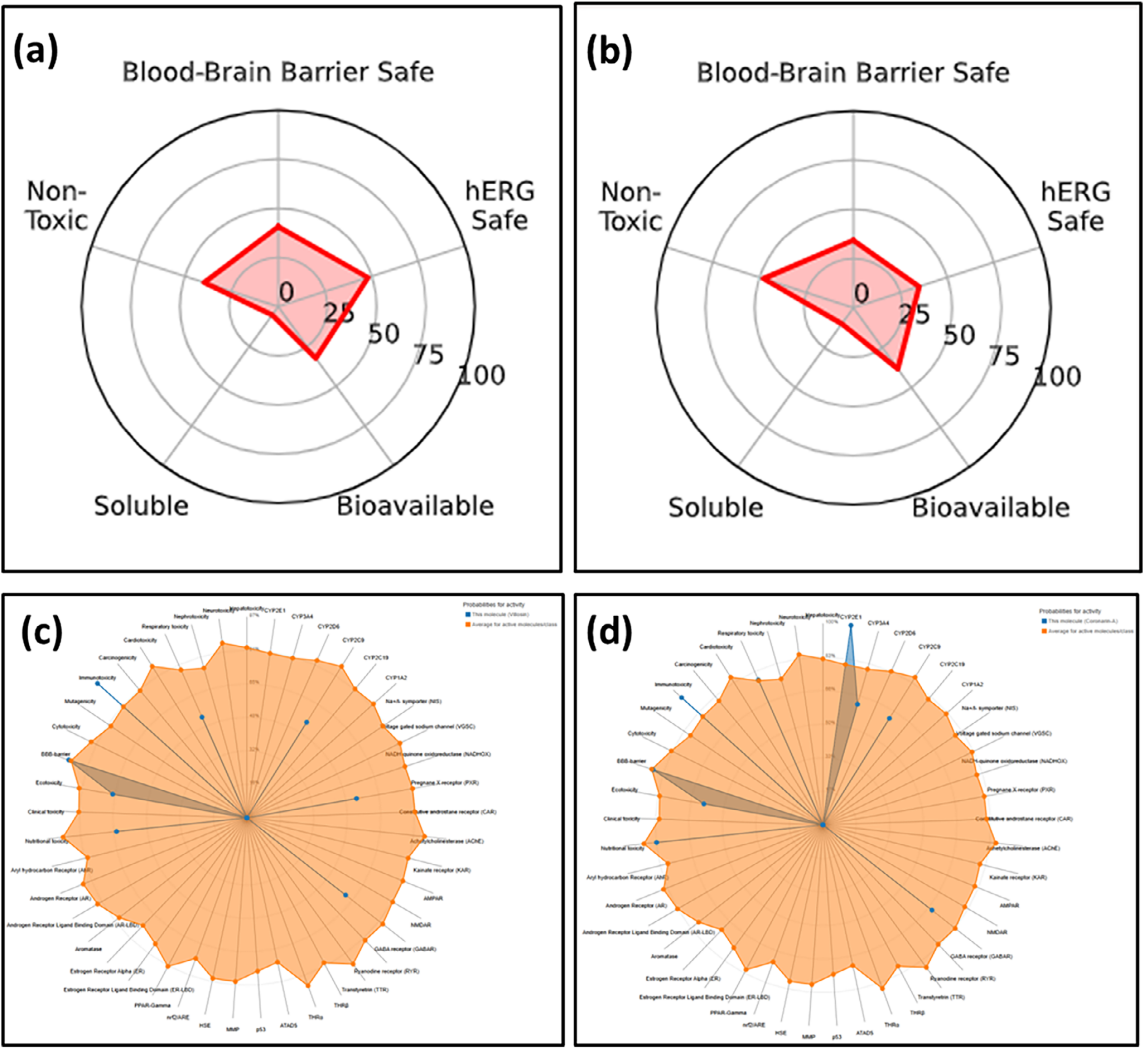
Radar chart for ADMET analysis of (a) compound 1, (b) compound 2; toxicity Web‐chart for ADMET analysis of (c) compound 1, (d) compound 2.

ADMET analysis, traditionally employed to evaluate pharmacokinetics in drug discovery, is equally significant in assessing the suitability of pesticidal compounds for human and environmental safety, ensuring minimal mammalian toxicity while maintaining efficacy [35]. In this study, the ADMET properties of the two flavonoids alongside their nematicidal activity against *Meloidogyne incognita* and acaricidal activity against *Oligonichus coffeae* were evaluated. The ADMET (Absorption, Distribution, Metabolism, Excretion, and Toxicity) analysis of the two labdane diterpenes coronarin A and villosin (Table 8) reveals encouraging pharmacokinetic and safety profiles that support their potential as eco‐friendly pest control agents. Both compounds exhibit favorable molecular characteristics, with compliance to Lipinski's Rule of Five, acceptable molecular weight (300.44 Da), and high human intestinal absorption, indicating good oral bioavailability. Coronarin A demonstrates a higher quantitative estimate of drug‐likeness (QED = 0.77) than Villosin (QED = 0.54), suggesting a better overall balance of bioactive properties. While both compounds display moderate aqueous solubility, their high PAMPA permeability and blood‐brain barrier penetration imply efficient systemic distribution. Notably, coronarin A shows a longer predicted half‐life (36.87 h) and greater metabolic stability, indicated by its moderate inhibition of CYP450 enzymes such as CYP2C19 and CYP2C9, which may contribute to prolonged pesticidal activity. In contrast, villosin exhibits slightly higher clearance rates, suggesting quicker metabolism but comparable bioavailability. Toxicological parameters also favor coronarin A, which presents lower mutagenicity, carcinogenicity, and hepatotoxicity scores than villosin, along with reduced risks of hERG channel inhibition and skin reactions. Both compounds demonstrate full plasma protein binding and safe profiles across various toxicity indicators, with predicted LD_50_ values placing them in low acute toxicity categories. Visual ADMET radar plots (Figure 9) further confirm these findings, portraying Coronarin A as more aligned with ideal bioactive compounds, while Villosin, though slightly less favorable in safety metrics, still exhibits strong pesticidal potential. Collectively, these insights highlight coronarin A and villosin as botanicals with balanced efficacy and safety for sustainable agricultural pest management.

**TABLE 8:** Table for ADMET analysis of compound 1 and compound 2.

Property	Coronarin A	Villosin
Molecular weight (Da)	300.44	300.44
LogP (log‐ratio)	5.06	4.82
Hydrogen bond acceptors	2.00	2.00
Hydrogen bond donors	1.00	0.00
Lipinski's rule of five	3.00	4.00
Quantitative estimate of drug‐likeness (QED)	0.77	0.54
Stereocenters	4.00	3.00
Topological polar surface area (TPSA) (A^2^)	33.37	26.30
Human intestinal absorption	1.00	1.00
Oral bioavailability	0.71	0.67
Aqueous solubility (log mol/L)	−5.38	−6.03
Lipophilicity (log‐ratio)	4.23	4.06
Hydration free energy (kcal/mol)	−6.38	−1.72
Cell effective permeability (log (10^−6^cm/s))	−4.63	−4.66
PAMPA permeability	0.98	0.98
P‐glycoprotein inhibition	0.49	0.42
Blood–brain barrier penetration	0.89	0.83
Plasma protein binding rate (%)	100.00	100.00
Volume of distribution at steady state (L/kg)	7.31	3.08
CYP1A2 inhibition	0.25	0.03
CYP2C19 inhibition	0.79	0.65
CYP2C9 inhibition	0.36	0.23
CYP2D6 inhibition	0.21	0.08
CYP3A4 inhibition	0.44	0.46
CYP2C9 substrate	0.27	0.13
CYP2D6 substrate	0.20	0.07
CYP3A4 substrate	0.64	0.69
Half‐life (h)	36.87	0.00
Drug clearance (hepatocyte) (µL/min/10^6^ cells)	57.79	75.62
Drug clearance (microsome) (µL/min/mg)	32.32	47.49
hERG blocking	0.52	0.32
Clinical toxicity	0.10	0.15
Mutagenicity	0.09	0.22
Drug induced liver injury	0.11	0.28
Carcinogenicity	0.09	0.19
Acute toxicity LD50	3.04	2.39
Skin reaction	0.63	0.92
Androgen receptor (full length)	0.05	0.06
Androgen receptor (ligand binding domain)	0.01	0.04
Aryl hydrocarbon receptor	0.16	0.01
Aromatase	0.45	0.29
Estrogen receptor (full length)	0.27	0.26
Estrogen receptor (ligand binding domain)	0.14	0.27
Peroxisome proliferator‐activated receptor gamma	0.02	0.08
Nuclear factor (erythroid‐derived 2)‐like 2/antioxidant responsive element	0.72	0.84
ATPase family AAA domain‐containing protein 5 (ATAD5)	0.01	0.03
Heat shock factor response element	0.50	0.61
Mitochondrial membrane potential	0.68	0.60
Tumor protein p53	0.04	0.15

The ADMET predictions generated using tools such as SwissADME provide valuable preliminary insights into the pharmacokinetic and toxicological profiles of coronarin A and villosin. However, these in silico models are primarily optimized for drug discovery and may not fully capture environmental or non‐mammalian toxicity endpoints relevant to pesticide regulation. Therefore, while the predicted low mammalian toxicity and favorable drug‐likeness are encouraging, they must be corroborated by comprehensive in vivo toxicological studies, ecotoxicological assessments, and environmental fate analyses before these compounds can be deemed safe for agricultural use.

## 3 Conclusion

This study successfully demonstrates the potent acaricidal and nematicidal efficacy of two labdane diterpenes, coronarin A and villosin isolated from the rhizomes of *Hedychium coronarium* (ginger lily). Through a combination of chromatographic purification, spectroscopic identification (FTIR, UV‐Vis, HPLC, and MS), and in vitro bioassays, both compounds exhibited significant concentration‐ and time‐dependent bioactivity against *M. incognita* (root‐knot nematode) and *O. coffeae* (red spider mite). Coronarin A showed superior nematicidal activity, on the other hand Villosin displayed stronger acaricidal effects, with a peak mite mortality of 90.28%. But activity against fungal pathogen *Pestalotiopsis microspora* was not too satisfactory. Only villosin showed activity against the target fungal pathogen but with a comparatively higher IC_50._ Molecular docking supported these bioactivities, with both compounds showing strong binding affinities to acetylcholinesterase (AChE) and GABA transaminase, confirming their neurotoxic modes of action. Notably, villosin exhibited a docking score of –10.0 kcal/mol against AChE, outperforming even the commercial standard fenpyroximate. Additionally, in silico ADMET profiling indicated favorable pharmacokinetics and low toxicity risks, with coronarin A demonstrating higher drug‐likeness and longer biological half‐life, while villosin offered slightly faster clearance. While the in vitro and in silico data presented here highlight the promising biocontrol potential of coronarin A and villosin, it is important to acknowledge the preliminary nature of these findings. The activities observed at concentrations in the mg/mL range suggest that further formulation optimization such as the development of nanoemulsions, synergistic combinations with other botanicals, or structural derivatization may be required to enhance bioavailability and reduce effective doses in field applications. Additionally, the scalability of isolation, cost‐effectiveness of production, and potential impacts on non‐target organisms and beneficial soil microbiota must be evaluated in subsequent studies. In the context of *Hedychium* derived terpenoids, coronarin A and villosin exhibit comparable or superior activity to previously reported labdane diterpenoids particularly against *M. incognita*, which reinforces the value of *H. coronarium* as a rich source of structurally diverse pesticidal compounds. However, field trials under realistic agronomic conditions are essential to validate the efficacy, persistence, and crop safety of these compounds before they can be integrated into commercial integrated pest management (IPM) frameworks. These findings position both compounds as candidates for the development of plant‐based biopesticides. However, further research is necessary to overcome practical challenges such as high effective concentrations, production scalability, and non‐target effects. Future work should focus on formulation development, field‐efficacy trials, and detailed environmental safety assessments to translate these preliminary results into sustainable, eco‐friendly pest management solutions.

## 4 Experimental Section

### 4.1 Plant Material

The medicinal and aromatic plant *Hedychium coronarium* was collected from Pantnagar in Uttarakhand, India. The plant material was identified by the plant taxonomist of the Department of Biological Sciences, G. B. Pant University of Agriculture and Technology, Pantnagar, Uttarakhand, India, and has been given voucher number GBPUH‐1035. The collected plant samples have been carefully preserved in the custody of the Department of Biological Sciences, G.B.P.U.A.&T., Pantnagar, for future reference.

### 4.2 Extraction

Fresh rhizomes of *Hedychium coronarium* were subjected to hydrodistillation for 3 h using a Clevenger‐type apparatus [36] to isolate the essential oils. The essential oils obtained were preserved separately for future research purposes and are not included in the present investigation. The primary objective of this step was to eliminate volatile constituents that might otherwise interfere with the analysis of other bioactive compounds, allowing a more accurate assessment of the pharmacological potential of the plant extracts, rather than that of its oleoresins.

After the essential oils were removed, the residual plant material was shade‐dried and finely powdered using a mechanical grinder. Organic solvents such as ethanol, methanol, acetone, hexane, and ethyl acetate are considered effective for extracting a wide range of phytoconstituents [37]. Briefly, the finely ground plant material (200 g) was immersed in DCM (400 mL) in airtight glass jars, ensuring that the DCM was sufficiently filled to cover the plant material. After every 3 days (approximately 72–75 h), the resulting supernatant was filtered through Whatman filter paper, and the remaining residues were used in subsequent extraction steps until the supernatant was colorless. Finally, a crude extract was obtained by evaporating the filtrates with a rotary evaporator under reduced pressure at 50°C. Again, 400 mL of chloroform was used for residue material (200 g) of rhizome per extraction cycle, and the process was repeated until the extract turned colorless, indicating exhaustive extraction. All the filtrates obtained from each solvent system were concentrated separately using a rotary evaporator under reduced pressure at 40°C to obtain the respective crude extracts [38]. The percent yield of each extract was calculated using the following equation:

$$Percent\;yield\;\left(\%\;DW\right)=\frac{\mathrm{Weight}\;\mathrm{of}\;\mathrm{dried}\;\mathrm{extract}}{\mathrm{weight}\;\mathrm{of}\;\mathrm{dried}\;\mathrm{plant}\;\mathrm{material}}\times 100$$

The resulting solvent extracts yielded 16.0 g (12.5%) of DCM extract and 44.9 g (5.98%) of chloroform extract. Both extracts were further subjected to stepwise chromatographic fractionation for purification and biological activities.

### 4.3 Isolation

Dichloromethane (DCM) extract of plant material weighing 16.0 g is used to start the procedure. First, 320.0 g of silica gel with a mesh size of 60–120 and 20% ethyl acetate (EtOAc) in hexane are used as the mobile phase in column chromatography of this extract. Fractions 40 through 67 are gathered in this stage. Re‐chromatography is done on 7.0 g of silica gel (mesh size 200–400) with 5% EtOAc in hexane, which is used to purify these fractions further, yielding fraction 23–40. Another round of re‐chromatography is performed on 7.0 g of silica gel (mesh size 200–400), using 0.5% EtOAc in hexane as the mobile phase and continuing the procedure. Fractions 28 to 37 obtained from this final step yielded compound 1.

The chloroform extract of *H. coronarium* rhizomes was mixed with 90 g of alumina to prepare a slurry, and over 300 g of the alumina column was eluted with chloroform till all material was eluted out. Concentrate and dry the eluted fraction using Rotavapor. The yield of the purified extract was found to be 10.68 g (1.424%). Purified extract dissolved in 20 mL of hexane and eluted over 60 g of 200–400 mesh silica column using hexane: Ethyl acetate: Acetone gradient (98:2, 96:2:2, 92:4:4). The eluted fraction was combined and concentrated, then purified, yielding 4.2 g (0.56%) of yellowish‐brown waxy compound 2.

### 4.4 Characterization of Bioactive Compounds

The bioactive fraction was chemically characterized by spectral analysis using HPLC, UV‐visible spectroscopy, FT‐IR, and mass spectrometry.

#### 4.4.1 Physical Property Assessment

The physical properties of compounds 1 and 2 were assessed through a series of standard observational and analytical techniques. Initially, the isolated compounds were examined for their color, appearance, and texture under natural light and using a stereomicroscope, providing preliminary insights into their physical form and crystallinity. Both compounds were then subjected to melting point determination using a digital melting point apparatus to evaluate their purity and compare against reference values; sharp and narrow melting ranges indicated a high degree of purity. Additionally, solubility tests were performed by dissolving small quantities of each compound in a range of solvents with varying polarities, including water, ethanol, methanol, chloroform, and the solubility behavior was recorded as soluble, partially soluble, or insoluble based on the extent of dissolution. These combined assessments supported the identification and characterization of compound 1 and compound 2.

#### 4.4.2 FT‐IR Analysis

FTIR analysis was conducted to identify the functional groups present in the isolated compounds. Characteristic absorption bands helped confirm the chemical moieties involved in compounds. FT‐IR spectra of the isolated compound were recorded in KBr pellets on a Perkin‐Elmer spectrophotometer and a Bruker Alfa II model. The FT‐IR spectrometer ranges from 4000 to 400cm^−1^ under transmission mode.

#### 4.4.3 Mass Spectrometry Analysis

Mass spectrometry was used to determine the molecular weight and structural information of the isolated compounds. This technique provided precise mass‐to‐charge (m/z) ratios for molecular identification. The mass spectra of the isolated compounds were acquired using a Waters Micromass Q‐Tof Micro mass spectrometer, equipped with ESI and APCI ionization sources. The hybrid Q‐ToF system operated over mass ranges of up to 4000 amu (quadrupole) and 20 000 amu (TOF). Samples were introduced at a flow rate of 0.05–5.0 mL/min. The instrument was calibrated for accurate mass determination and molecular weight confirmation.

#### 4.4.4 HPLC Analysis

The purity of the isolated compounds was assessed using a Waters Alliance HPLC system equipped with an auto‐injector and operated via Empower 3 software. Chromatographic analysis was carried out on an Eclipse Plus C_18_ column (150 mm × 4.6 mm, 5 µm particle size). The mobile phase consisted of acetonitrile (ACN) and water containing 0.1% ortho‐phosphoric acid (OPA) in a ratio of 70:30 (v/v). The flow rate was maintained at 0.7 mL/min, with a column temperature of 25°C. The total run time for both analyses was 30 min. Sample injections were performed automatically, and chromatograms were monitored to evaluate retention time and peak purity.

#### 4.4.5 UV‐Vis Analysis

UV–visible spectrophotometric analysis was performed using a Thermo Scientific Genesis 10S UV‐Visible spectrophotometer. Samples were scanned in the range of 200–800 nm using quartz cuvettes (path length 1 cm) with appropriate solvent blanks as reference. The absorption maxima (λmax) were recorded for qualitative characterization.

The resulting spectral profiles and chromatographic behaviors were critically analyzed and compared with those reported in the existing scientific literature for the respective compounds. This comparative approach enabled the confirmation of the chemical identities of the isolated constituents, ensuring consistency with previously characterized reference compounds.

### 4.5 Evaluation of In Vitro Biological Activities

#### 4.5.1 Acaricidal Activity

Mites were reared on tea plant (*Camellia sinensis*) leaves in the Department of Entomology, Faculty of Agriculture, UBKV, West Bengal. Rearing conditions were 26°C ± 2°C and 65% ± 5% R.H. with a 12 h light and 12 h dark photoperiod throughout the year to prevent diapause induction. Water and tea leaves were changed every 2 days [39].

**FIGURE 10:**
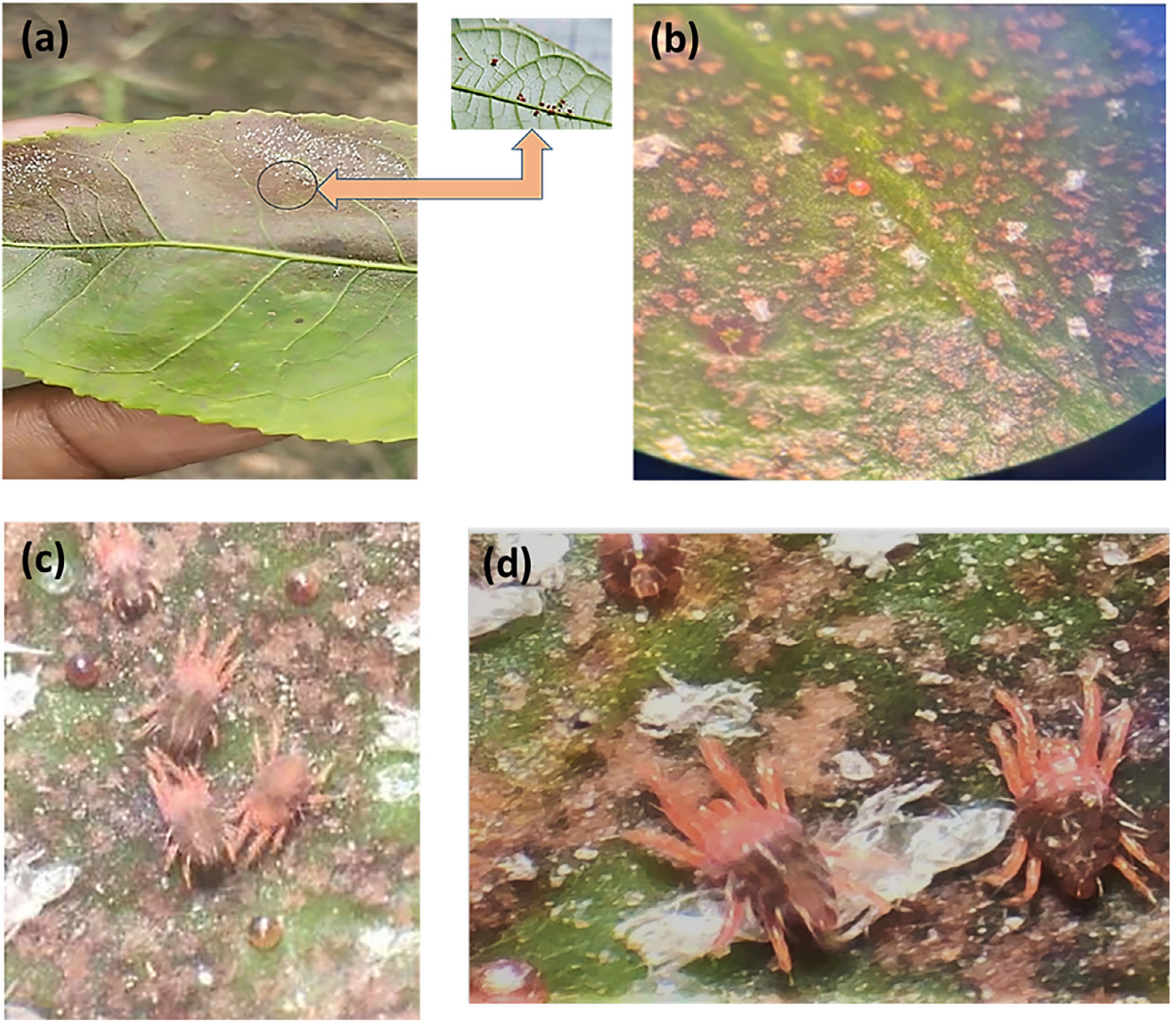
(a) Damaging effect with adults on tea leaf of *Oligonychus coffeae*; (b) nymphs and eggs; (c) adult mites; and (d) adult mites with eggs.

The leaf disc method [31] was implemented for evaluation of the acaricidal activity against adult females (Figure 10) of *O*. *coffeae* for the preliminary screening; an appropriate amount (0.1 g) of compound 1 was dissolved in DCM (2 mL) and diluted to 100 mL with distilled water containing 0.05% Tween 80 for a final concentration of 1.0 mg/mL, and the same amount of compound 2 was dissolved in 10 mL of 20% methanol. Leaf discs of 6 cm diameter were prepared and placed with their ventral surface down over the wet cotton taken in a petri plate (9 cm diameter), and each disc represents a replicate. Thirty adult mites were released on each disc with a brush and allowed to settle in the disc. Three replicates were maintained for each treatment. Individual petri dishes were examined under a stereo binocular after 24, 48, and 72 h of treatment for counting the live and dead mites. The adult mortality at different intervals was subjected to ANOVA to infer about the difference among the treatments at *p* < 0.05 for variance using the least significant difference. Untreated controls containing 0.05% Tween 80 were considered for all samples, except for compound 2, for which 20% methanol was used as the control. If mortality in the controls ranged between 5% and 20%, Abbott's formula was applied to correct it [40]. Three replicates of each treatment were tested. Based on the initial test results, different concentrations (1.0, 3.0, 5.0 mg/mL) were tested for each compound for the determination of the median lethal concentration (LC_50_) according to the probit analysis [41].

Adult mortality rate was calculated as:

$$Mortality\;\%=\frac{\mathrm{Dead}\;\mathrm{mites}}{\mathrm{Total}\;\mathrm{number}\;\mathrm{of}\;\mathrm{mites}}\times 100$$

#### 4.5.2 Nematicidal Activity

Eggs of *Meloidogyne incognita* were extracted from tomato (*Solanum lycopersicum*) roots infected with the nematode collected from THE Directorate of Research, UBKV, West Bengal, in a glasshouse maintained at 25°C ± 2°C. The sample was collected based on the visual symptoms of root‐knots or galls formed in the tomato plant. Hand‐picked mature egg masses (Figure 2) from infected roots were cultured in distilled water in a growth chamber at 25°C. Emergent juveniles were collected and stored at 5°C for further use [42, 43].

Within 48 h of hatching, 100 second‐stage juveniles (Figure 2) were removed from the eggs and placed on gridded petri dishes containing 1.0 mL of stock solution and distilled water as part of the in vitro mortality test. The first isolated compound 1, is dissolved in 20% MeOH, while the compound 2 dissolved in a small amount of chloroform in 10% Tween 20 to create three samples with varying concentrations (1.0, 3.0, and 5.0 mg/mL).

**FIGURE 11:**
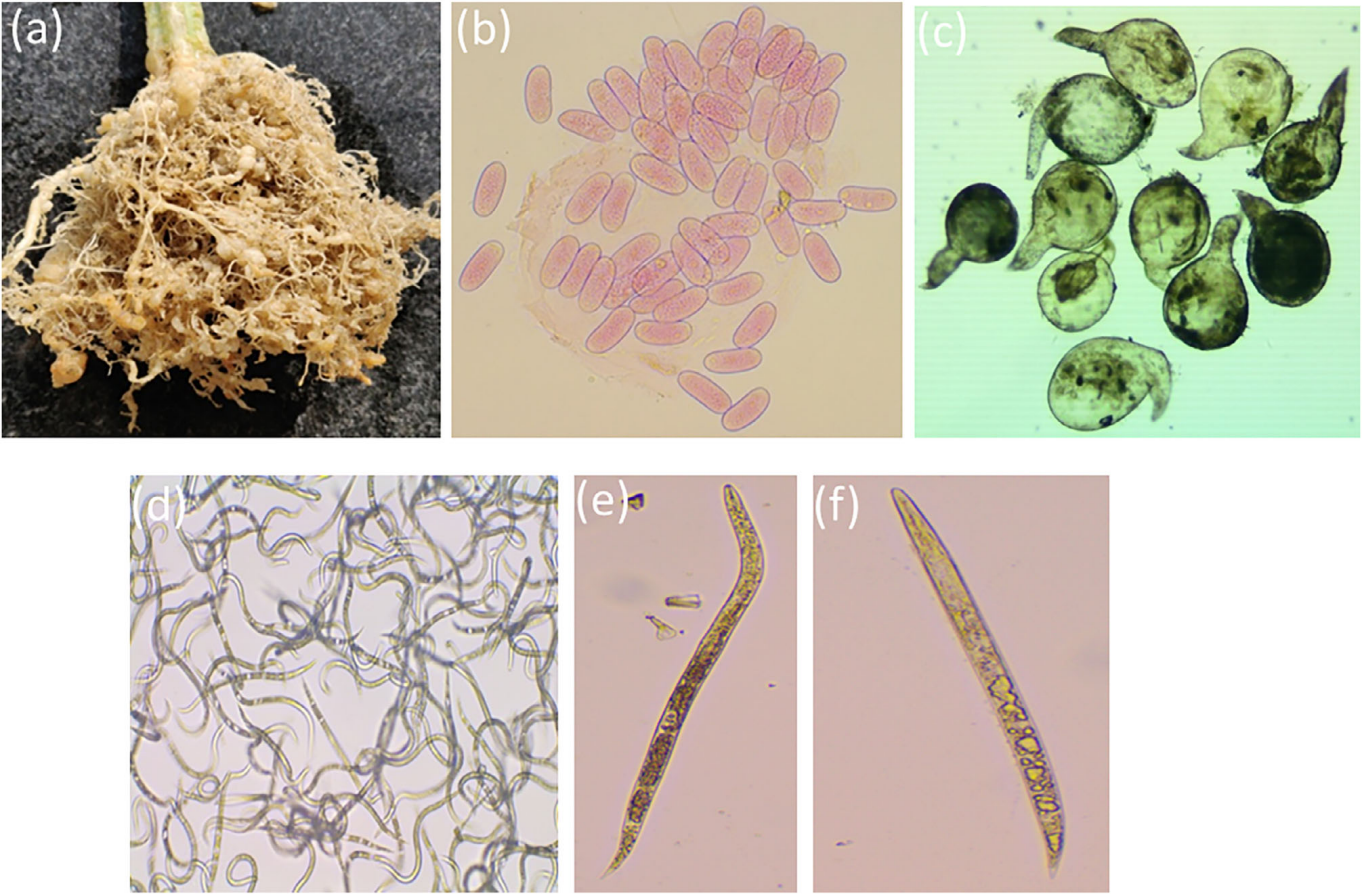
*Meloidogyne incognita*: (a) root knot nematode infected plant, (b) eggs, (c) adult females, (d) pure population of second‐stage juveniles, (e) second‐stage juvenile, and (f) dead second‐stage juvenile.

In triplicate, the treatments were administered in a randomized order. The control group consisted of the juveniles submerged in 20% methanol and Tween 20. Using a stereo‐binocular microscope, the number of dead juveniles was counted throughout 24, 48, and 72 h. Nematodes in the Petri plate that were completely still (dead larvae) (Figure 11) were removed and submerged in distilled water.

Abbott's formula was used to determine the percentage of deaths [40]. The LC_50_ value was analyzed by probit analysis. The concentration that kills 50% of the test population (LC_5_₀) was reported in µg/mL, consistent with the units used for the compound concentrations in biological activities:

$$Mortality\;\%=\frac{\mathrm{Nt}-\mathrm{Nc}}{100\%-\mathrm{Nc}}\times 100$$

where *N*_t_ = mortality in treatment, and *N*_c_ = mortality in control.

The egg hatching inhibition assay, similar to the mortality assay but with nematode eggs (approximately 100 eggs per 1 mL solution), recorded egg hatching at the same time intervals. The percentage of egg hatching inhibition was calculated as:

$$\begin{array}{ccc} & & \%\;egg\;hatching\\ & & =\frac{\mathrm{Egg}\;\mathrm{hatching}\;\mathrm{in}\;\mathrm{control}-\mathrm{Egg}\;\mathrm{hatching}\;\mathrm{in}\;\mathrm{treatment}}{\mathrm{Egg}\;\mathrm{hatching}\;\mathrm{in}\;\mathrm{control}}\times 100 \end{array}$$

#### 4.5.3 Antifungal Activity

The in vitro antifungal activity of isolated compounds was evaluated using the agar disc diffusion method, as described by [44]. *Pestalotiopsis microspora*, the fungal species, was obtained from the Department of Plant Pathology, College of Agriculture, G.B. Pant University of Agriculture and Technology (G.B.P.U.A.&T.), Pantnagar. The fungal isolates were reactivated by culturing them on potato dextrose agar (PDA) medium. This was done by aseptically transferring mycelial discs into sterile Petri dishes containing the PDA medium. The cultures were then incubated under controlled conditions at 25°C ± 2°C for a duration of 7 days to ensure optimal fungal growth and viability prior to antifungal screening.

A 7‐day‐old culture of *Pestalotiopsis microspora* was utilized to prepare standardized mycelial inoculum discs for evaluating the antifungal efficacy of Compound 1 and Compound 2. Sterile potato dextrose agar (PDA) plates were prepared, and autoclaved filter paper discs impregnated with three concentrations (40, 60, and 80 µL/mL) of each test compound were aseptically placed at equidistant margins of the Petri plates.

Simultaneously, mycelial discs of *P. microspora* were inoculated at separate peripheral locations on each plate to ensure adequate contact with the treated zones. For control treatments, PDA plates were inoculated solely with fungal discs and sterile filter paper discs moistened with distilled water, omitting the test compounds.

All experimental plates were incubated at 26°C ± 2°C with 68% ± 2% relative humidity until the mycelial growth in the control group reached the edge of the petri dish. Antifungal efficacy was assessed by measuring the radial zone of mycelial growth inhibition (in millimeters) around each treated disc.

The percentage inhibition of mycelial growth was calculated according to the formula [45]:

$$\%\;Inhibition=\frac{\left(X-Y\right)}{x}\times 100$$

where *X* = Mycelial growth in control (mm)

*Y* = Mycelial growth in treatment (mm)

### 4.6 In Silico Study

#### 4.6.1 Molecular Modeling Study

Molecular docking studies were conducted to investigate the binding modes of the isolated compounds with the crystalline acetylcholinesterase (AChE) enzyme structure of *Meloidogyne incognita*, given AChE's critical role in synaptic transmission and movement regulation in this nematode. The protein structure of AChE was retrieved from the RCSB Protein Data Bank (PDB) using the corresponding PDB ID: 6XYU. GABA transaminase (GABA‐T) is targeted by acaricidal compounds because it plays a crucial role in GABA metabolism, which is essential for neurotransmission in pests such as mites. Inhibiting GABA‐T disrupts GABA degradation, leading to excessive neuronal inhibition and paralysis, making it an effective target for pest control. The protein structure of GABA‐T was retrieved from the RCSB Protein Data Bank (PDB) using the corresponding PDB ID: 1OHW. For antifungal activity the protein structure of translation elongation factor 1‐alpha from *Pseudopestalotiopsis theae* was retrieved from AlphaFold database. Translation elongation factor 1‐alpha (TEF1‐α) is a universally conserved protein that plays a central role in protein biosynthesis. In fungi, including the genus *Pestalotiopsis*, TEF1‐α not only facilitates translation but also serves as a valuable molecular marker for phylogenetic and taxonomic studies due to its moderately conserved nature across fungal taxa.

For ligand structures, 3D structures of the compounds coronarin A and villosin were obtained from the PubChem database in Structural Data File (SDF) format. Physostigmine (CID: 5983) was selected as the standard reference inhibitor for AChE to benchmark docking scores. Using PyRx software's integrated Open Babel tool, these SDF files were imported, where energy minimization was performed through charge addition and optimization under the universal force field. Subsequently, ligands were converted to AutoDock Ligand format (PDBQT) for docking. Docking was carried out in PyRx using the Vina Wizard tool to evaluate binding affinities and receptor‐ligand interactions. The Vina Wizard interface allowed for protein and ligand selection, and docking was initiated by executing the “Run Vina” function. Docking results were analyzed and exported in CSV format via the “Analyze Vina” tool. Visualization of 2D and 3D binding interactions was achieved through Biovia Discovery Studio‐2023 Client, enhancing the interpretability of docking poses and receptor‐ligand interactions.

#### 4.6.2 ADMET Prediction

ADMET (Absorption, Distribution, Metabolism, Excretion, and Toxicity) prediction is pivotal in pesticide and biopesticide research, offering early insight into the safety and efficacy profiles of candidate compounds. By identifying molecules with optimal pharmacokinetic and toxicological properties, ADMET modeling significantly reduces the time, cost, and failure rate during development. In silico tools also help mitigate environmental impact and non‐target organism toxicity, supporting the creation of eco‐friendly crop protection agents. Furthermore, ADMET predictions facilitate regulatory approval by providing essential pre‐screening data, thereby advancing the development of sustainable agrochemicals. For the current study, the chemical structures of the pure compounds were obtained from PubChem (https://pubchem.ncbi.nlm.nih.gov/) and imported in SMILES format for computational analysis. ADME properties, including gastrointestinal absorption, blood‐brain barrier penetration, lipophilicity, and solubility, were evaluated using the Swiss ADME online tool (http://www.swissadme.ch/).

### 4.7 Statistical Analysis

Experiments were conducted in triplicate, with data presented as mean ± standard deviation. Statistical analysis was performed using two‐way ANOVA followed by Tukey's test (*p* < 0.05) in OriginPro2022b to identify significant differences. Two‐way ANOVA evaluates the main effects of two independent factors and their interaction effect, but it does not reveal which specific group means differ from each other. Tukey's honestly significant difference (HSD) test addresses this as a post hoc procedure by conducting all pairwise comparisons of means, such as between levels of one factor or across factor combinations, while controlling the family‐wise error rate to prevent inflation due to multiple testing.

## Author Contributions

Riddhiman Lahiri contributed to conceptualization, methodology design, investigation, formal analysis, and the preparation of the original draft. Ravendra Kumar supervised the project, administered overall coordination, and contributed to reviewing and editing the manuscript. Sushila Arya was involved in methodology development and validation. Alok Pratap Singh contributed to the methodology and investigation. Sakshi Negi was involved in formal analysis and other necessary editing. Shivang Joshi was responsible for data curation and formal analysis. Pooja Bargali contributed to data curation and formal analysis. Suraj N. Mali was involved in methodology and supervision. Shilpi Rawat provided experimental facilities for anti‐fungal activity. Mozaniel Santana de Oliveira contributed to reviewing and editing the manuscript. Prahlad Sarkar provided experimental facilities and supervision for acaricidal and nematicidal activity.

## Conflicts of Interest

The authors declare no conflicts of interest.

## Data Availability

The data that support the findings of this study are available from the corresponding author upon reasonable request.

## References

[1] B. Deka , A. Babu , C. Baruah , and S. Sarkar , “Plant Extracts as Potential Acaricides for the Management of Red Spider Mite, *Oligonychus coffeae* Nietner (Acarina: Tetranychidae), in the Tea Ecosystem: An Eco‐friendly Strategy,” Frontiers in Agronomy 4 (2022): 685568, 10.3389/fagro.2022.685568.

[2] A. Bhujel , M. A. H. O. N. A. R. Singh , M. Choubey , and M. Singh , “Pest and Diseases Management in Darjeeling Tea,” International Journal of Agricultural Science and Research 6 (2016): 469–472.

[3] A. Barua , A. Babu , and R. C. Rajkhowa , “Seasonal Abundance and Predatory Potential of Stethorusaptus Kapur (Coleoptera: Coccinellidae): A Biocontrol Agent of Tea Red Spider Mite *Oligonychus coffeae* Nietner (Acarina: Tetranychidae),” Proceedings of the Zoological Society 71 (2016): 224–228, 10.1007/s12595-016-0197-4.

[4] D. Sundararaju and P. C. Sundara Babu , “ *Helopeltis* spp. (Heteroptera: Miridae) and Their Management in Plantation and Horticultural Crops of India,” Journal of Plantation Crops 27 (1999): 155–174.

[5] L. K. Hazarika , M. Bhuyan , and B. N. Hazarika , “Insect Pests of Tea and Their Management,” Annual Review of Entomology 54 (2009): 267–284, 10.1146/annurev.ento.53.103106.093359. 19067632

[6] S. A. Rau , “Note on Thrips and Mites of Tea in Southern India,” Planters' Chronology 60: 486–491.

[7] L. R. Jeppson , H. H. Keifer , and E. W. Baker , Mites Injurious to Economic Plants (University of California Press, 1975), 10.1525/9780520335431.

[8] A. K. Pandey , G. D. Sinniah , A. Babu , and A. Tanti , “How the Global Tea Industry Copes With Fungal Diseases—Challenges and Opportunities,” Plant Diseases 105 (2021): 1868–1879, 10.1094/PDIS-09-20-1945-FE. 33734810

[9] S. D. Joshi , R. Sanjay , U. I. Baby , and A. K. A. Mandal , “Molecular Characterization of *Pestalotiopsis* spp. Associated With Tea (*Camellia sinensis*) in Southern India Using RAPD and ISSR Markers,” Indian Journal Biotechnology 8 (2009): 377–383.

[10] Z. H. Wang , Z. X. Zhao , N. Hong , et al., “Characterization of Causal Agents of a Novel Disease Inducing Brown‐Black Spots on Tender Tea Leaves in China,” Plant Diseases 101 (2017): 1802–1811, 10.1094/PDIS-04-17-0495-RE. 30676920

[11] P. F. Carvalho , “Pesticides, Environment, and Food Safety,” Food Energy Security 6, no. 2 (2017): 48–60, 10.1002/fes3.108.

[12] S. Arya , R. Kumar , O. Prakash , et al., “Therapeutic Bioactivities and Chemical Composition Analysis of Rhizome Oleoresin of *Hedychium coronarium* Collected From Uttarakhand, India,” Combinatorial Chemistry and High Throughput Screening 28, no. 18 (2024): 3224–3237, 10.2174/0113862073327439241119113953. 39843450

[13] J. S. Raut and S. M. Karuppayil , “A Status Review on the Medicinal Properties of Essential Oils,” Industrial Crops and Products 62 (2014): 250–264, 10.1016/j.indcrop.2014.05.055.

[14] B. Singh , S. K. Borthakur , and S. J. Phukan , “A Survey of Ethnomedicinal Plants Utilized by the Indigenous People of Garo Hills With Special Reference to the Nokrek Biosphere Reserve (Meghalaya), India,” Journal of Herbs Spices & Medicinal Plants 20, no. 1 (2014): 1–30, 10.1080/10496475.2013.819476.

[15] L. Chen , Z. Wen , P. Sung , et al., “New Labdane‐type Diterpenoid and Cytotoxic Constituents of *Hedychium coronarium* ,” Chemistry of Natural Compounds 53 (2017): 72–76, 10.1007/s10600-017-1914-6.

[16] A. S. Lima , H. N. P. C. Junior , L. M. Costa‐Junior , O. S. Monteiro , J. G. S. Maia , and C. Q. da Rocha , “Anthelmintic Effect of Essential Rhizome Oil From Hedychium Coronarium Koenig (Zingiberaceae) Introduced in Northeastern Brazil,” Acta Tropica 218 (2021): 105912, 10.1016/j.actatropica.2021.105912. 33826931

[17] F. P. Gullo , A. M. Fusco‐Almeida , M. J. S. Mendes‐Giannini , et al., “Essential Oils and Major Compounds of *Hedychium coronarium* Koenig (Zingiberaceae) Against Pathogenic Yeast of *Candida* and *Cryptococcus* Genus,” Revista de Ciências *Farmacêuticas Básica e Aplicada* 37 (2016): 1.

[18] S. Arya , R. Kumar , S. Joshi , et al., “Seasonal Variation in the Composition of Hedychium Coronarium Oleoresins and Their Screening as Potential Biopesticides,” Plant Biosystems 159, no. 2 (2025): 289–301, 10.1080/11263504.2025.2466562.

[19] S. Arya , R. Kumar , O. Prakash , M. Latwal , G. Pandey , and S. K. R. Srivastava , “Chemical Composition, Nematicidal, Insecticidal and Herbicidal Activities of *Hedychium coronarium* J. Koenig Rhizome Oleoresin,” Pharma Innovation Journal 11, no. 6 (2022): 761–766.

[20] S. Arya , S. K. Mahawer , H. Karakoti , et al., “Study of the Variability of the Chemical Profile, and Biological Activity Approaches of *Hedychium coronarium* J. Koenig Essential Oil From Different Habitats of Uttarakhand, India,” Journal of Food Quality 1 (2025): 7335134.

[21] N. Chimnoi , B. Sritularak , V. Lipipun , and K. Likhitwitayawuid , “Labdane‐type Diterpenes From the Rhizomes of *Hedychium coronarium* ,” Journal of Natural Products 71, no. 7 (2008): 1193–1196.18558743

[22] J. J. Chen , C. W. Ting , Y. C. Wu , et al., “New Labdane‐type Diterpenoids and Anti‐Inflammatory Constituents From *Hedychium coronarium* ,” Journal International Molecular Sciences 4, no. 7 (2013): 13063–13077, 10.3390/ijms140713063. PMC3742174 23799360

[23] H. Karakoti , P. Bargali , R. Kumar , et al., “Comparison of Chemical Composition, Nematicidal Activity and Acetylcholinesterase Inhibition With In Silico Mechanistic Insights of Hedychium Flavescens Essential Oils and Extracts From Aerial Parts and Rhizomes,” Biochemical Systematics and Ecology 121 (2025): 104987, 10.1016/j.bse.2025.104987.

[24] P. Pachurekar and A. K. Dixit , “A Review on Pharmacognostical Phytochemical and Ethnomedicinal Properties of *Hedychium coronarium* J. Koenig an Endangered Medicine,” International Journal of Chinese Medicine 1, no. 2 (2017): 49–61, 10.11648/j.ijcm.20170102.13.

[25] Z. Hu , X. Liu , M. Tian , et al., “Recent Progress and New Perspectives for Diterpenoid Biosynthesis in Medicinal Plants,” Journal of Medical Research and Review 41, no. 6 (2021): 2971–2997, 10.11648/j.ijcm.20170102.13. 33938025

[26] F. N. Taveira , A. B. Oliveira , J. D. Souza Filho , and F. C. Braga , “Epimers of Labdane Diterpenes From the Rhizomes of *Hedychium Coronarium* J. Koenig,” Revista Brasileira Farmacognosia 15 (2005): 55–59, 10.1590/S0102-695X2005000100012.

[27] H. Itokawa , H. Morita , I. Katou , et al., “Cytotoxic Diterpenes From the Rhizomes of *Hedychium coronarium* ,” Planta Medica 54, no. 4 (1988): 311–315, 10.1055/s-2006-962442. 3222377

[28] S. Arya , R. Kumar , H. Karakoti , et al., “Pesticide and Antimicrobial Evaluation of Three Labdane‐Type Diterpenes, and One Flavonoid Glycoside, Isolated From Rhizomes of *Hedychium coronarium* J. Koenig,” Natural Product Research 39 (2024): 5418–5424, 10.1080/14786419.2024.2413035. 39377434

[29] I. Kumrit , A. Suksamrarn , P. Meepawpan , S. Songsri , and N. Nuntawong , “Labdane‐Type Diterpenes From *Hedychium gardnerianum* With Potent Cytotoxicity Against Human Small Cell Lung Cancer Cells,” Phytotherapy Research 24, no. 7 (2010): 1009–1013, 10.1002/ptr.3057. 19960422

[30] P. Xiao , C. Sun , M. Zahid , O. Ishrud , and Y. Pan , “New Diterpene From Hedychium Villosum,” Fitoterapia 72, no. 7 (2001): 837–838, 10.1016/S0367-326X(01)00307-0. 11677028

[31] E. H. Siegler , “Leaf‐Disk Technique for Laboratory Tests of Acaricides,” Journal of Economic Entomology 40, no. 3 (1947): 441–442, 10.1093/jee/40.3.441a. 20344769

[32] M. E. I. Badawy , S. A. El‐Arami , and S. A. M. Abdelgaleil , “Acaricidal and Quantitative Structure Activity Relationship of Monoterpenes Against the Two‐Spotted Spider Mite, *Tetranychus urticae* ,” Experimental and Applied Acarology 52 (2010): 261–274, 10.1007/s10493-010-9363-y. 20431924

[33] T. Shen , S. J. He , H. Y. Yang , G. L. Li , J. L. Xu , and Y. L. He , “Two Novel Diterpenoid Alkaloids From Aconitum Pendulum,” Chemistry and Biodiversity 21, no. 8 (2024): e202400977, 10.1002/cbdv.202400977. 38837616

[34] R. Kaomongkolgit , K. Jamdee , S. Wongnoi , N. Chimnoi , and S. Techasakul , “Antifungal Activity of Coronarin D Against Candida albicans,” Oral Surgery, Oral Medicine, Oral Pathology, and Oral Radiology 114, no. 1 (2012): 61–66, 10.1016/j.oooo.2012.01.010. 22727093

[35] S. Sarkhel and G. R. Desiraju , “N‐H…O, O‐H…O, and C‐H…O Hydrogen Bonds in Protein–Ligand Complexes: Strong and Weak Interactions in Molecular Recognition,” Proteins: Structure, Function, and Bioinformatics 54 (2004): 247–259, 10.1002/prot.10567. 14696187

[36] G. Moroy , V. Y. Martiny , P. Vayer , B. Villoutreix , and M. A. Miteva , “Toward In Silico Structure‐Based ADMET Prediction in Drug Discovery,” Drug Discovery Today 17, no. 1 (2012): 44–55, 10.1016/j.drudis.2011.10.02. 22056716

[37] J. F. Clevenger , “Apparatus for the Determination of Volatile Oil,” Journal of the American Pharmacists Association 17, no. 4 (1928): 345–349, 10.1002/jps.3080170407.

[38] B. Sultana , F. Anwar , and M. Ashraf , “Effect of Extraction Solvent/Technique on the Antioxidant Activity of Selected Medicinal Plant Extracts,” Molecules (Basel, Switzerland) 14 (2009): 2167–2180, 10.3390/molecules14062167. 19553890 PMC6254218

[39] S. K. Mahawer , R. Kumar , H. Karakoti , et al., “Phytochemistry and Potential Nematicidal, Phytotoxic and Antibacterial Effects of *Alpinia malaccensis* (Burm. f.) Roscoe (Zingiberaceae) and Molecular Dockign Study,” Journal of Chemistry Africa 8 (2025): 1–23, 10.1007/s42250-025-01247-7.

[40] W. S. Abbott , “A Method of Computing the Effectiveness of an Insecticide,” Journal of Economic Entomology 18, no. 2 (1925): 265–267, 10.1093/jee/18.2.265a.

[41] D. J. Finney , Probit Analysis, 3rd ed., (Cambridge University Press, 1971), ISBN: 9780521080415, 10.4236/ce.2012.326134.

[42] J. D. Eisenback , “Detailed Morphology and Anatomy of Second‐Stage Juveniles, Males, and Females of the Genus *Meloidogyne* (Root‐Knot Nematodes),” An Advanced Treatise on Meloidogyne 1 (1985): 47–77, https://www.researchgate.net/publication/233858354.

[43] S. Maher , R. Salam , F. Mukhttar , N. Khan , and F. Batool , “Nematicidal Effect of Methanolic Plant Extracts Against the Root‐Knot Nematode, *Meloidogyne incognita* ,” Pakistan Journal of Nematology 32, no. 2 (2024): 187–195, 10.17582/journal.pjn/2024/42.2.120.126.

[44] P. Bargali , R. Kumar , A. Devrani , et al., “Essential Oils Work Synergistically to Mitigate Pathogenic Impact of *Meloidogyne Incognita*, *Rhizoctonia Solani* and *Sclerotinia Rolfsii* ,” Biocatalysis and Agricultural Biotechnology 58 (2024): 103160, 10.1016/j.bcab.2024.103160.

[45] H. Abd‐El‐Khair and G. El‐Gamal Nadia , “Effects of Aqueous Extracts of some Plant Species Against *Fusarium Solani* and *Rhizoctonia solani* in *Phaseolus vulgaris* Plants,” Archiv *für Phytopathologie und Pflanzenschutz* 44, no. 1 (2011): 1–16, 10.1080/03235400802678436.

